# Dysphagia with Anæmia, with Reports on Five Cases

**Published:** 1932

**Authors:** E. Watson-Williams

**Affiliations:** Surgeon-in-Charge Ear, Nose and Throat Department, Bristol Royal Infirmary


### DYSPHAGIA WITH ANÆMIA, WITH REPORTS OF FIVE CASES.

BY

E. Watson-Williams, M.C., Ch.M., F.R.C.S.,

*Surgeon-in-Charge Ear, Nose and Throat Department, Bristol Royal Infirmary.*

---

The syndrome to which we now assign this non-committal title is of no great rarity but of some importance, were it only because the first diagnosis is often "hysteria." More than fifty years ago Weir-Mitchell drew attention to the intractable nature of "hysterical dysphagia," and the tendency of these patients to develop a profound anæmia was observed even earlier than this. When this anæmia becomes manifest, with pigmentation and achlorhydria, a diagnosis of pernicious anæmia is suggested, while the cracks at the angles of the mouth, and the chronic glossitis may lead us astray by simulating a syphilitic condition.

*Clinical Picture.*—The patients are all women, and, what is more, generally women of middle age, thin, pale and "worried." Advice is usually sought on account of difficulty in swallowing, progressive over months or years, which has gradually reduced the victim to a fluid diet. The food "sticks in the swallow," so that the patient chokes and has to bring it up ; once it can be got to enter the gullet there is no further difficulty. There may be considerable variation from day to day in the degree of obstruction.

indicating an element of spasm. Sometimes if the first mouthful can be forced down there is no further trouble. There is little wasting, but complaints of feelings of suffocation, wind, palpitation, etc., are common, strongly suggesting a neurosis. The anæmia is usually of long duration, the complexion "earthy," and often there is pigmentation in addition; but on investigation the first proves to be of secondary type with a low hæmoglobin percentage, a colour index about 0·7, and some anisocytosis. The mucous membranes of the lips, mouth and pharynx are thin, pale, dry and sore; the cracks at the angles of the mouth, already mentioned, are commonly found; and the tongue, although free from fissures, is partly or wholly denuded of papillæ, glossy and dry. There is no history of syphilis or miscarriages and the Wassermann test is negative. Many of the patients are edentulous, some show enlargement of the spleen. Achlorhydria or oligochlorhydria is usual. The swallowing of a barium feed under X-rays may reveal some hesitation at the commencement of the act of swallowing, but once the feed has entered the gullet its further progress is normal. Direct examination of the œsophagus may show either a spasmodic contraction or an actual constriction of the upper end, below which it is normal; in either case the atrophy and fragility of the mucosa is conspicuous.

*Cases.*—The five cases reported below are relatively early, and were diagnosed on clinical grounds. They do not, therefore, show either the duration of symptoms or such advanced pathological changes as some that have been recorded.

(1) S.L., aged 49. May, 1932, complains of difficulty in swallowing solids during one year; the food "catches in the swallow," and she usually has to bring it up, so that now she can only take fluids, and even these pass very slowly. She is getting thinner, but not much ; always thin. All her life she has been anæmic, and has suffered from indigestion and wind, but never before had throat trouble. No pain. All symptoms are getting much worse, she is " always tired." The difficulty in swallowing varies, " comes and goes " ; but there is now at all times quite considerable difficulty.

On examination a thin woman, very pale, with an " earthy " complexion, and some darkening of the skin at the sides of the neck and ribs and over the knees. Lips very pale ; the tongue shows large areas smooth and devoid of papillæ, but no cracks. At the angles of the mouth are well-marked cracks ; inner surfaces of cheeks pale and sore, palate waxy yellow. Only lower incisor teeth present, some retraction of gums. T. 97·8°. P. 64. Spleen not enlarged.

Blood : Hb. 60 per cent. ; R.B.C., 4,000,000 ; C.I., 0·7 ; W.B.C., 7,000. " The red cells stain comparatively well ; anisocytosis and poikilocytosis are moderate, no megalocytes ; no nucleated reds ; no abnormal white cells." W.R., negative. Hæmolysis begins in 0·42 per cent. saline, complete in 0·32 per cent.* Van den Bergh negative, direct and indirect. Test meal : no free acid in fasting fluid or first four specimens ; at two and a half, three, and three and a half hours free acid 0·022%,* combined 0·11%.† Barium bolus : some hesitation at entrance to œsophagus, otherwise normal. Direct laryngoscopy : the opening of the œsophagus is a transverse slit 5 mm. across with apparently a post-cricoid web of mucous membrane round the posterior margin, and just below this a similar anterior web. The mucosa very pale, thin and fragile, without the longitudinal folds usually associated with spasm ; despite care in dilatation, the mucosa over the back of the cricoid split. Large tube passed to the cardia ; normal œsophagus with very pale mucosa, no spasm.

Treatment : Pil. ferri. gr. x. t.d.s., later gr. xv. t.d.s. June, 1932.—" Much better " (looks it) ; the swallowing now normal for all ordinary solids. July 27th.—The anæmia is not noticeable ; discharged to her own doctor to continue iron.

(2) Sarah P., aged 47. May, 1932, complains of " bad throat," excess of phlegm and difficulty in getting it up, with pain on attempting to swallow solids, although she can always get these down with an effort ; symptoms have been present

* *i.e.* Normal. † Reckoned as HCl.

during several months. She used to get suffocative attacks, during which the throat closed up and she felt she could not breathe. For many years she has suffered from palpitation and wind; she has always been anæmic (not gravely), not getting worse.

On examination a thin, pale woman, manifestly "neurotic." The complexion is distinctly earthy, with dark pigmentation of the neck. The lips are very pale, there is a deep crack at each angle of the mouth, the mucous membrane of the cheeks and soft palate is pale, thin and "dry," that of the nose is "rather dry," and the back wall of the pharynx "very dry"; larynx normal: edentulous. T. 97°. P. 72.

Blood: Hb. 50 per cent.; R.B.C. 4,900,000; C.I. 0·5. "The red cells show a bigger central-poorly-staining area than usual; definite anisocytosis, many red cells being smaller than normal; except for microcytes everything else appears normal." Fragility of corpuscles; hæmolysis begins in 0·42 per cent. and is complete in 0·32 per cent. saline. W.R. negative. Van den Bergh negative, direct and indirect. Test meal: fasting and first two specimens, no free HCl; at one and a half hours a trace, and at two hours a small trace.

This patient did not wish for any investigation of the dysphagia; she agreed to allow examination, and was admitted for the purpose, but the ward had to be closed on account of infection; she was sent to the Out-Patient Department, taking gr. xl. daily of the pil. ferri. This so much improved her that a month later she declined any further examination; after three months the complexion was good, although still somewhat pale, the cracks had disappeared and the wind, palpitation and general health were very much improved; "the nerves have improved enormously."

(3) Annie B., aged 44. August, 1931, complains of nine months' difficulty in swallowing, all solid food tending to stick at the Adam's apple; she can only take fluids. She has for many years been anæmic, and this is tending to get worse; the digestion is poor, appetite good. She has always been thin, but has definitely lost weight recently.

On examination a small, thin, "worried," very pale woman—the pallor is remarkable—but no pigmentation is noted. Lips quite white, deep cracks at angles of the mouth; mucosa of interior of mouth pale and sore; tongue perfectly smooth, sore and dry; pharyngeal mucosa dry and very thin; teeth good; spleen normal; temperature subnormal.

Blood: Hb. 65 per cent.; R.B.C. 3,600,000; C.I. 0·9; fragility normal (1932). W.R. negative. Test meal: complete absence of free HCl in all specimens. Barium bolus; some gulping and hesitation in the pharynx, spasm of cardia, no dilation or other abnormality.

Direct œsophagoscopy: the mucosa is remarkably pale; well-marked spasm of the entrance of the œsophagus which yielded to the passage of a large tube; below this the œsophagus was quite normal, the tube passing through a normal cardia into the stomach.

This patient ceased to attend; she returned in April, 1932, when the swallowing was quite good for all food. But the anæmia was even more conspicuous, although she had gained several pounds in weight; she was unable to sleep. Pil. ferri., gr. xl. daily was prescribed. By July swallowing, sleep and digestion were quite satisfactory, the cracks of the mouth had healed, the soreness had disappeared (although the tongue was still smooth), and there was no obvious anæmia; she continues under treatment.

(4) Florence D., aged 46. April, 1932, complains that during the last five months food lodges in the throat, and she can now only take fluids or quite soft food. "Once it starts it goes down all right." At night she gets a feeling of suffocation in the neck; she has always been thin, but since the trouble started has lost weight rapidly. Four years ago she choked on her food; even before then she had "a small swallow," and had to be very particular in mastication. Since that time she has been even more so, but in spite of this the difficulty in swallowing has at last driven her to seek assistance. She has been anæmic all her life, but manages to get about and do her work, though tired on any extra exertion; the anæmia is not getting worse.

On examination a small, thin woman of remarkable pallor, the complexion earthy, no pigmentation. Lips very pale, definite crack at each corner of the mouth; mucosa of mouth pale and sore; whole tongue is smooth, glossy, "dry-looking" and sore, without cracks; pharyngeal mucosa thin and pale; edentulous; spleen not enlarged; temperature subnormal.

Blood: Hb. 70 per cent.; R.B.C. 4,400,000; C.I. 0·7. "The red cells do not stain well; there is moderate anisocytosis, no megalocytes, no microcytes, no nucleated reds." Hæmolysis begins in 0·42 per cent. saline, is complete in 0·30 per cent.

W.R. negative. Test meal; no free HCl in fasting fluid or any of the specimens. Barium bolus (not observed) reported normal.

Direct œsophagoscopy: the œsophageal opening is very small (=circle of 4 mm. diam.) and tight; in passing the speculum the anterior wall was lacerated—"apparently an œsophageal web." Below the constriction the œsophagus was normal but pale.

Treatment: gr. lx. daily of the pil. ferri.

Three months later she was quite different. Swallowing was comfortable for everything except crusts; digestion good, no evidence of anæmia; cracks and soreness of mouth have disappeared, but tongue still smooth.

(5) Dorothy A., aged 42. September, 1932, complains that she cannot swallow solids. Eighteen months ago she could not take meat, and for the last three weeks she can take no solids at all. "It is like a tight band across the top of my throat when I swallow." The food sticks, but by an effort it could at first often be forced down; not intermittent. She feels low, has always been thin, and her own doctor thinks she has recently got thinner, but she has not noticed this; she can do her ordinary housework. She has been slightly anæmic for seven years. General health, digestion, sleep, "nerves," good.

On examination a rather pale, tall, slight woman; the complexion is clear, no evidence of wasting, lips and conjunctiva good colour. Insides of lips sore (six weeks), small crack at each angle of mouth; tongue has lost papillæ along either margin, not in centre. Edentulous. Granular pharyngitis, mucosa normally pink; nose, larynx normal. T. 96°.

Blood: Hb. 65 per cent.; R.B.C. 3,200,000; C.I. 1·0; W.B.C. 5,000 (normal differential count). "Red cells show anisocytosis, poikilocytosis and increased central vacuolation; halometer reading 4·5°; size of corpuscles=7·64$\mu$." Fragility normal; W.R. and Van den Bergh negative. Although not the face, the body is very thin; no pigmentation, spleen normal. Test meal; well-marked *excess* of free HCl and total acid in fasting fluid and all subsequent specimens; *e.g.* free HCl at half hour=0·15 per cent., at one hour 0·25 per cent., at two and a half hours 0·33 per cent. Barium feed; some holding up of the feed behind larynx (which suggested to Dr. Mayes a small pouch), otherwise normal. Direct œsophagoscopy: mucosa pink; the opening of the gullet at first appears normal; but on attempting to pass tube, although there is no trace of spasm there is a firm web limiting the lumen to 10 mm. diameter, running semi-circularly round posterior wall behind cricoid; slight force dilated it with just a trace of bleeding. Lower down the œsophagus shows a rather red mucosa, and near each end patches of whitish "sodden" epithelium, *i.e.* œsophagitis; cardia and stomach normal. Following the dilatation she can swallow normally, and has begun to take iron.

Clinically this case is typical, except that the anæmia is not conspicuous; the hæmoglobin figure is unexpectedly characteristic, although the C.I. is high; the marked hyperchlorhydria is in contradiction with the usual findings. The œsophagitis is an unusual feature; the hypopharyngeal "band" affords an insight into the causation of the dysphagia, but an element of spasm must be postulated to account for the degree of difficulty present.

*Comment.*—The ætiology of this condition is obscure. D. R. Paterson[1] and A. Brown Kelly, who first drew attention to the actual nature of the œsophageal changes, definitely consider these primary, and the anæmia secondary to interference with nutrition. "At a meeting of this section in 1919 Brown Kelly[2] and I drew attention to a clinical syndrome in women, the essential feature of which is a gradually increasing inability to swallow, due to spasm of the pharyngo-œsophageal sphincter; often associated with atrophic changes in the buccal and pharyngeal mucosa, and at a *later stage with anæmia*, which is to be regarded as a *secondary manifestation* of the dysphagia. Plummer, approaching it from the physician's point of view, called it hysterical dysphagia, and later Vinson[5] laid stress on the anæmia and splenomegaly. . . . Such patients are sometimes labelled hysterical, often incorrectly, and if they appear unduly nervous they are only observing a caution that the dread of choking imposes. . . . The blood changes are those of chlorosis and secondary anæmia with considerable reduction in the hæmoglobin (Munro Cameron)."[6] The patients are women and come under observation in middle life with a long history of gradually increasing dysphagia, which eventually confines them to a fluid diet. Mild glossitis with atrophy may be early ; later the tongue is smooth and the buccal mucosa dry and pale. (The pharyngeal atrophy secondary to atrophic rhinitis does *not* lead to dysphagia.) Anæmia is a *late* symptom of gradual development. The spleen may be enlarged. Malignant disease is often a late development of this syndrome.

On the other hand, it has been suggested that the anæmia is the primary condition of which the changes in the alimentary tract are sequelæ. Dr. R. S. Johnson[3,7] has reported six cases of dysphagia with anæmia, under Dr. G. Graham and Professor F. R. Fraser at St. Bartholomew's Hospital. All were females, aged 33 to 48. The anæmia was marked, of secondary type, hæmoglobin 32 to 50 per cent., C.I. 0·5. The complexion is striking, brownish-yellow, suggesting pernicious anæmia. Glossitis and cracks at the angles of the mouth were noted in four ; dysphagia was severe in two, mild in three, none in one. Œsophagoscopy : two normal, pallid mucosa in one, an area of œdema bleeding readily in one, impossible on account of spasm in one. All showed complete achlorhydria ; only one (without dysphagia) slight enlargement of spleen. The others showed increased fragility of corpuscles, hæmolysis beginning in 0·55 per cent. saline and being complete in 0·45 per cent. He suggests initial specific anæmia with increased fragility, and achlorhydria, and *secondary* changes in mucous membranes producing dysphagia.

My own cases definitely support the view that the anæmia precedes the dysphagia. Further, in Case 3 relief of the dysphagia during eight months (so that the weight increased) brought no improvement in the anæmia ; while in Case 2 treatment of the anæmia seems to have had a beneficial influence on the dysphagia. On the other hand, I have been unable to confirm any increase in the fragility of the red cells. This might be explained on the ground that the anæmia in my cases was much less marked than in those of Dr. Graham ; yet it was of long duration in all five, so that this slight increase in fragility can hardly be an essential feature. Again, although Dr. Graham's cases showed profound anæmia, in only two was the dysphagia severe. Also, it seems at least possible that in other cases a certain degree of anæmia has long existed without attracting much attention, until the progress of the dysphagia forces the victims to seek advice ; and perhaps actual interference with nutrition produces a conspicuous increase in a previously mild anæmia.

In fact, one is tempted to suggest that the actual clinical syndrome presents three main features: dysphagia, anæmia and stomatitis or glossitis ; and that of these three any one may for a time hold the field, until either its increased severity or the effects of one of the others drives the sufferer to seek relief. So that the case may appear with a long history of increasing dysphagia and recent anæmia, or as long continued anæmia and subsequent dysphagia, or even as a stomatitis with difficulty in swallowing as a complication. (Indeed, on reference to my notes I have found two cases of sufferers from "sore mouth," of whom both complained of some trouble in swallowing and one had definite anæmia.) It seems probable that some toxic or perhaps dietetic factor* is responsible for the whole of the changes observed. But as to the nature of that factor we are still quite in the dark.

*Treatment.*—This is simple. The dysphagia is relieved by the passage of a large tube, a procedure which demands some care owing to the fragility of the mucous membranes. The anæmia is best treated by large doses of inorganic iron, gr. xl. to gr. lx. of the pil. ferri daily; this has proved much more efficacious than organic iron or liver extract, etc.[4] The medicinal treatment needs to be continued for four to six months at least, and the patient should be kept under observation for two years, in case there is any recurrence of dysphagia (treated by bougies) or other trouble. The observation of Paterson on the subject of malignant change demands attention.

*Summary* :—

1. The clinical features of "dysphagia with anæmia" are described.
2. Five cases are reported in detail.
3. The ætiology and treatment are discussed.

I am indebted to Dr. A. D. Fraser for the pathological reports cited.

REFERENCES.

[1] *Proc. Roy. Soc. Med.* (*Section of Laryngology*), 1930–31, xxiv. 1,203.

[2] *Ibid.*, 1918–19, xii. 235.

[3] *Ibid.*, 1930–31, xxiv. 1,206.

[4] *Ibid.* (*Section of Therapeutics*), 1930–31, xxiv. 543 (with bibliography).

[5] "Hysterical Dysphagia," *Minnesota Med.*, 1922, v. 107.

[6] *Quart. Jour. Med.*, 1928, xxii. 43.

[7] *Ibid.*, 1932, N.S., i. 41.

* To be quite *dernier cri* one ought to postulate a hypothetical allergic state.

